# In silico analysis of aqueous root extract of *Rotula aquatica* Lour for docking analysis of the compound 3-*O*-acetyl-11-keto-β-boswellic acid contents

**DOI:** 10.1186/s40064-016-3134-0

**Published:** 2016-09-05

**Authors:** Bhavaniamma Vijayakumari, Venkatachalam Sasikala, Singanallur Ramu Radha, Hiranmai Yadav Rameshwar

**Affiliations:** 1 Department of Botany, Avinashilingam University for Women, Coimbatore, Tamil Nadu 6410043 India; 2 School of Natural Resources Management and Environmental Sciences, College of Agriculture and Environmental Sciences, Haramaya University, P.O. Box #337, Dire Dawa, Ethiopia

**Keywords:** Docking analysis, *Rotula aquatica*, Antiurolithiatic activity, Tamm–Horsfall protein

## Abstract

Molecular docking is a bioinformatics tool used to study and analyse ligand receptor interactions. This helps in identifying the receptors (molecular targets) for different ligands. Using these technologies, compound isolation and drug discovery from herbals is achieved. Herbs are widely used in treatment of various ailments from time immemorial. Phytochemists and drug developers are now interestingly working in developing new molecules that can act effectively than conventional drugs. As they are developing it mostly from herbs they are found to be effective and safer drugs and quantity to be used become minimum. *Rotula aquatica* Lour is a plant distributed widely in India and used for urinary disorders. The plant root was extracted and studied for its active compounds that possess antiurolithiatic activity. After performing various preliminary phytochemical studies and applying chromatographic methods, molecular docking was carried out with isolated bioactive compound and Tamm–Horsfall protein (THP). By docking analysis the bioactive compound 3-*O*-acetyl-11-keto-β-boswellic acid interacted with THP and it may inhibit calcium oxalate crystallization.

## Background

Molecular docking is one of the in silico method which is more efficient compared to in vitro and in vivo method for its capability of finding the active compound in medicinal plants. A three dimensional structure becomes very important in the molecular docking methods that depicts the compound. During this era of advancements the study and documentation of structural compounds from medicinal plants are important (Yanuar et al. 2011). Utilisation of computers and softwares are leading to the increased computing capabilities that provide opportunities to develop simulations and calculations in drug design. This method includes a structure based drug design and ligand based drug design. In the field of structure based drug design molecular docking is commonly used to predict and inter molecular complex between the drug molecules with its target protein. For this a set of data that contains information on the ligand and or drug to be docked and protein targets to be used are needed. The structure of the ligand and protein should be three dimensional (Hawkins and Skillman 2011; Abraham 2003).

Tamm–Horsfall (THP) is one of the main components of urinary protein. It is a glycoprotein produced and secreted by the thick ascendant limb of the loop of Henle, being the abundant protein in normal human urine, excreted in quantities of 20–200 mg/24 h (Hoyer and Seiler 1979; Kumar and Muchmore 1990). THP of normal subjects inhibits the aggregation but has little effect on nucleation and growth of CaOx crystals (Worcester et al. 1988). However, THP activity is influenced by its own concentration, urinary pH and ionic strength, playing a dual role such as inhibitor as well as promoter in crystal formation depending on the environmental conditions (Hess 1994). Moreover, THP isolated from the urine of recurrent stone formers sometimes becomes a promoter of CaOx aggregation due to tendency to self-aggregation, which removes it from effective interaction with CaOx monohydrate crystals (Muchmore and Decker 1985).

Medicinal plants are advantageous in the field of drug discovery as they are utilized by humans for centuries. The bioactive compounds found in the plants are having many properties that are applied in the treatment of diseases. Molecular docking was used to study the interaction of withanolides with DNA binding site of NF-kB through docking analysis (Nithya et al. 2009). *Rotula aquatica* Lour is a plant commonly used for its antiurolithiatic activity in traditional herbal treatment system, The Ayurveda. The preliminary phytochemical analysis of aqueous root extract of *R. aquatica* revealed the presence of various secondary metabolites like alkaloids, flavanoids, phenols, saponins, tannins, terpenoids, anthraquinones, anthocyanin, protein and carbohydrates (Vijayakumari et al. 2013). Hence, further HPTLC, GC–MS analyses were carried out in root extract to assess the active compounds with bioactive properties. The unknown organic compounds in a complex mixture can be determined by interpretation and also by matching the spectra with reference spectra. The compound was characterized by IR, ^1^H NMR, ^13^C NMR and mass spectral techniques. Further, the compound isolated from aqueous extract of *R. aquatica* roots were used for molecular docking studies. In the present era of bioinformatics, Molecular docking is one of the effective techniques to predominant binding modes of the ligand with the protein of known three-dimensional structure. Studies on binding modes are essential to elucidate key structural characteristics interaction and they provide helpful data for designing effective inhibitors. In order to identify antagonist for urinary stones molecular docking was carried out with entry level receptor namely Tamm–Horsfall protein in the present study.

Docking is a method of molecular modeling, which predicts the preferred orientation of one molecule to a second when bound to each other to form a stable complex. Molecular docking can be defined as an optimization problem, which would describe the “best-fit” orientation of a ligand that binds to a particular protein of interest and is used to predict the structure of the intermolecular complex formed between two or more molecules. The most interesting case is the protein ligand interaction, because of its applications in medicines. Ligand is a small molecule, which interacts with protein’s binding sites. There are several possible mutual conformations in which binding may occur. These are commonly called binding modes. In modern drug designing, molecular docking is routinely used for understanding drug information about drug receptor interactions and is frequently used to predict the binding orientation of small molecule drug candidates to their protein targets in order to predict the affinity and activity of the small molecule. The present paper deals with the utility of compound isolated from *R. aquatica* aqueous root extract by molecular docking to assess its antiurolithiatic property with THP.

## Methods

Molecular docking of bioactive compound isolated from *R. aquatica* aqueous root extract was carried out with Tamm–Horsfall protein (THP) using automated docking software.

### LigPrep

LigPrep is a robust collection of tools designed to prepare high quality, all-atom 3D structures for large number of drug-like molecules, starting with 2D or 3D structures in sdf or Maestro format. The resulting structures can be saved in either sdf or Maestro format. The simplest use of LigPrep produces a single, low energy, 3D structure with correct chiralities for each input structure with various ionization states, tautomers, stereochemistries, ring conformations and eliminate molecules using various criteria including molecular weight or specified numbers and types of functional groups present. The LigPrep script provides an efficient way to use a set of tools for ligand preparation collectively and consistently.

### Protein preparation

Since, 3D structure of Tamm–Horsfall protein was not available in the protein structural data base, a homology model of the THP with a sequencing number P07911 was generated using modBase automodel based on the template 3qw9A. The active site of the target protein was indentified using Schrodinger module. Glide calculations use an all-atom force field for accurate energy evaluation. Thus, Glide requires bond orders and ionization states to be properly assigned and performs better when side chains are reoriented when necessary and steric clashes are relieved. The entire procedure can be performed in the Protein Preparation Wizard panel, from the Workflows menu on the main toolbar.

### Receptor grid generation

Glide searches for favourable interactions between one or more ligand molecules and a receptor molecule, usually a protein. The 3-*O*-acetyl-11-keto-β-boswellic acid ligand binds with relatively hydrophobic amino acids includes GLY 534, GLY 590, PHE 587, PRO 528. The binding pocket are lined by the remaining amino acids namely GLN 524, HIS 529, ASP 525, ARG 526, ARG 583, ARG 586, SER 583, SER 589, CYS 527, THR 585. The shape and properties of the receptor are represented on a grid by several different sets of fields that provide progressively more accurate scoring of the ligand poses. The options in each tab of the receptor grid generation panel allows to define the receptor structure by excluding any co crystallized ligand that may be present, determine the position and size of the active site as it will be represented by receptor grids, and set up Glide constraints.

Ligand docking jobs cannot be performed until the receptor grids have been generated. Receptor grid generation requires a “prepared” structure: an all atom structure with appropriate bond orders and formal charges. The receptor grid generation panel has three tabs which is used to specify settings for the receptor grid generation job:

- Receptor
- Site
- Constraints

### Glide 5.9 (glide-based ligand docking with energetics)

Glide offers the full spectrum of speed and accuracy from high throughput virtual screening of millions of compounds to extremely accurate binding mode predictions, providing consistently high enrichment at every level. glide searches for favourable interactions between one or more ligand molecules and a receptor molecule, usually a protein. Each ligand must be a single molecule, while the receptor may include more than one molecule, e.g., a protein and a cofactor. Glide can be run in rigid or flexible docking modes; the latter automatically generates conformations for each input ligand. The combination of position and orientation of a ligand relative to the receptor, along with its conformation in flexible docking, is referred to as a ligand pose. The ligand poses that glide generates pass through a series of hierarchial filters that evaluate ligands interaction with the receptor. The initial filters test the spatial fit if the ligand to the defined active site, and examine the complementarity of ligand-receptor interactions using a grid based method patterned after empirical Chem Score function.

Poses that pass the screens enter the final stage of the algorithm, which involves evaluation and minimization of a grid approximation to the OPLS-AA non-bonded ligand interaction energy. Finally, the minimized poses are re-scored using Schrodinger’s proprietary GlideScore scoring function. GlideScore is based on ChemScore, but includes a steric-clash term and adds buried polar terms devised by Schrodinger to penalize electrostatic mismatches, and has modifications to other terms.

$${\text{GScore}}\, = \,0.065\,\times\,{\text{vdW}}\, + \,0.130\,\times\,{\text{Coul}}\, + \,{\text{Lipo}}\, + \,{\text{Hbond}}\, + \,{\text{Metal}}\, + \,{\text{BuryP}}\, + \,{\text{RotB}}\, + \,{\text{Site}}$$

### Ligand docking

Glide ligand docking jobs require a set of previously calculated receptor glides and one or more ligand structures. The force field used for docking is the OPLS_2001 force field. Typically, Glide standard-precision docking is used to find probable good binders in a large set; the top-scoring 10–30 % can then be investigated more intensively using glide extra-precision (XP) docking or other methods available from Schrodinger.

The ligand docking panel has five tabs.

- Settings
- Ligands
- Constraints
- Similarity
- Output

### Ligplot

It is a program for automatically plotting protein–ligand interactions. It automatically generates diagrams of protein–ligand interactions for a given PDB file. The interactions shown are those mediated by hydrogen bonds and by hydrophobic contacts. Hydrogen bonds are indicated by dashed lines between the atoms involved.

## Results and discussion

**Fig. 1:**
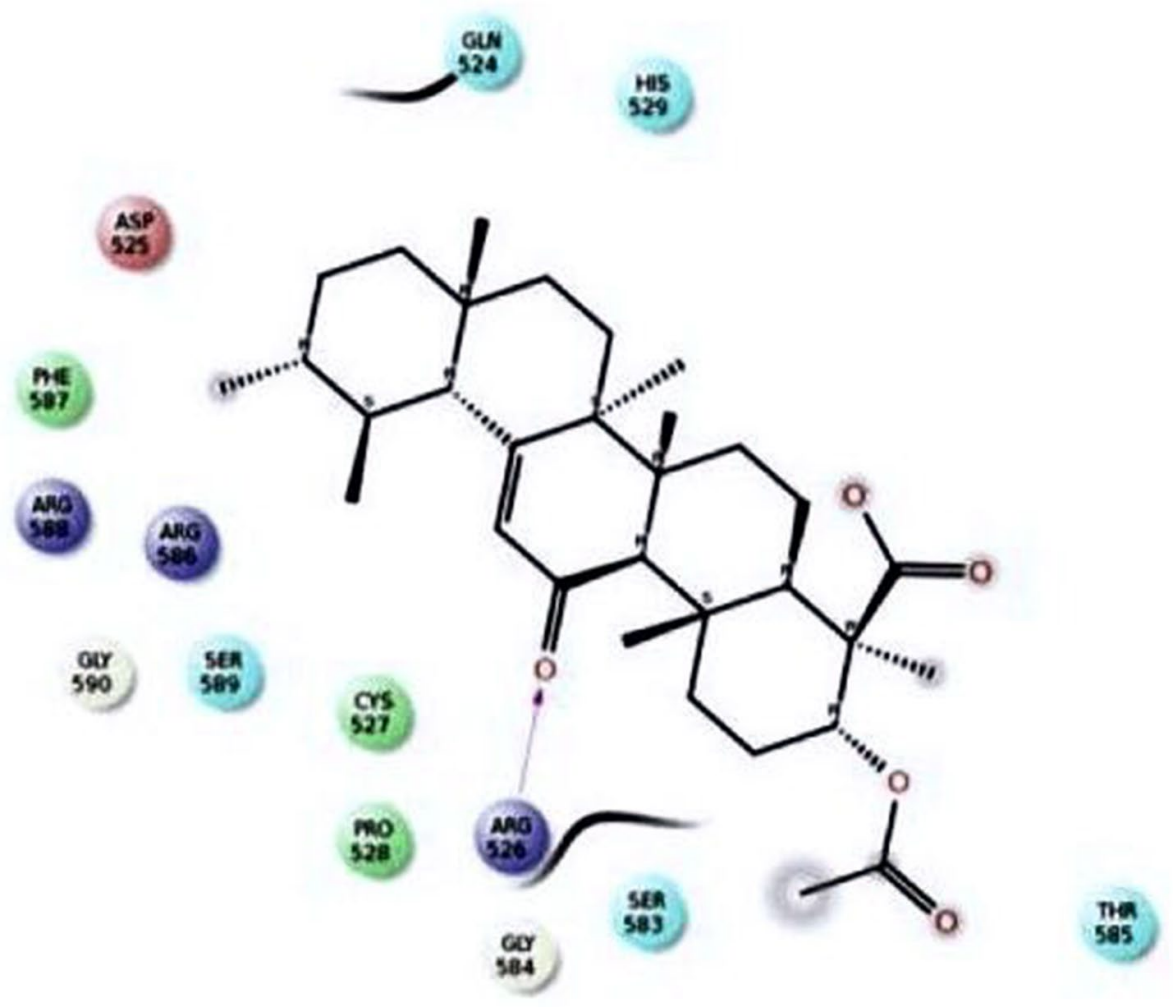
LigPlot view of the docked structure

**Fig. 2:**
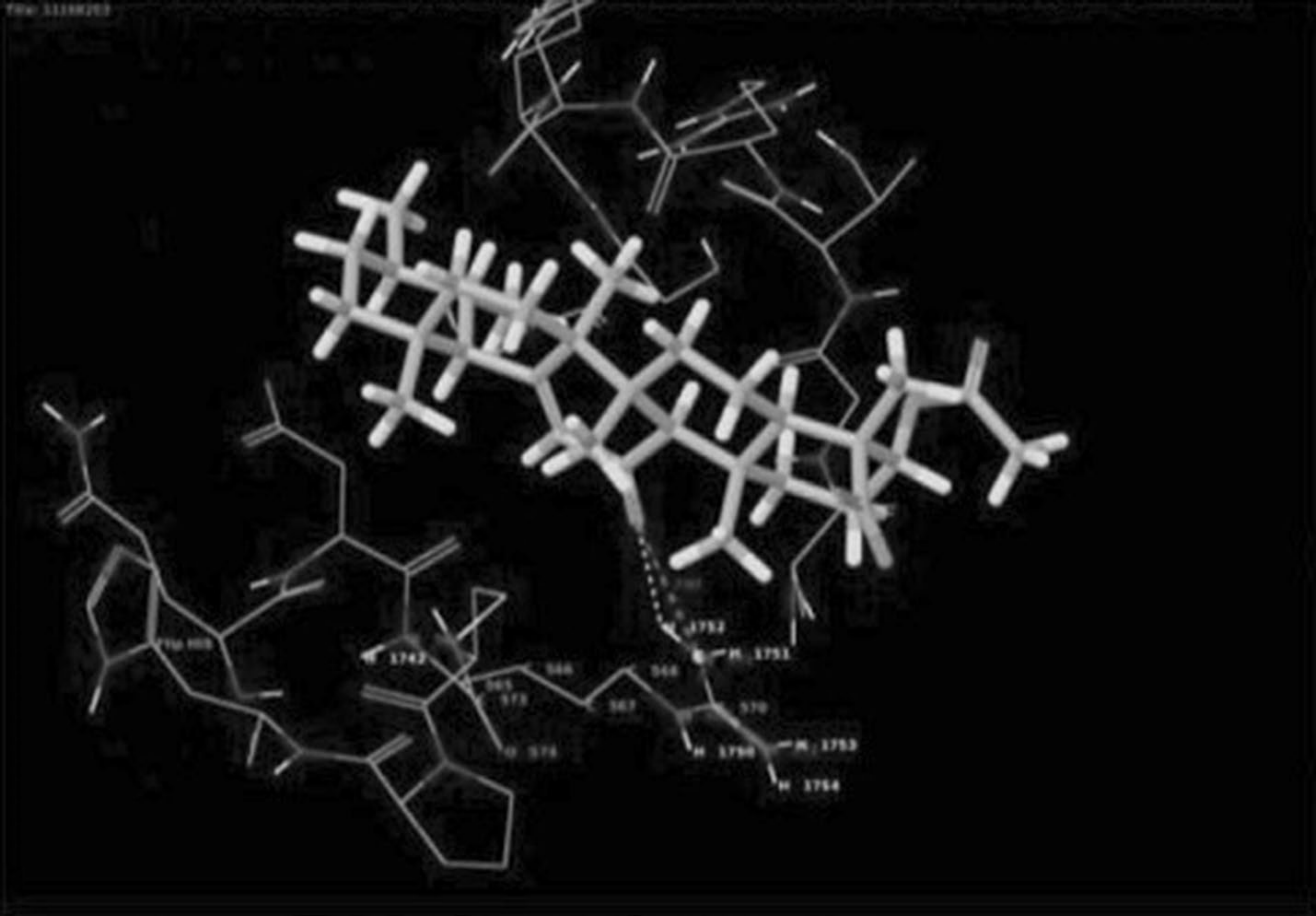
Molecular docking of Tamm–Horsfall protein with 3-*O*-acetyl-11-keto-β-boswellic acid and its inhibition

 The histopathological analysis of rat models have shown reduced calcium oxalate depositions and other abnormalities in *R. aquatica* Lour root treatment that shows the utility in treatment of urolithiasis. It is mainly considered with the dissolution of existing stones and preventing recurrence of stones. The bioactive compound Triterpenoid was isolated from the aqueous root extract of *R. aquatica* and it was characterised by employing different chromatographic and spectral techniques. Performance of docking analysis with the compound 3-*O*-acetyl-11-keto-β-boswellic acid from *R. aquatica* with Tamm–Horsfall protein exhibited the efficiency of interaction. The interaction brings an idea that the compound may inhibit calcium oxalate crystallization in urolithiatic condition (Figs. 1, 2).

### Molecular docking

Molecular docking is an efficient technique to predict the predominant binding modes of the ligand with the protein of known three-dimensional structure. Studies on binding modes are essential to elucidate key structural characteristics interaction and they provide helpful data for designing effective inhibitors.

The glide score, which distinguishes molecules based on their interacting ability, was calculated for the ligand. The least glide score or energy was more effective. The active compound 3-*O*-acetyl-11-keto-β-boswellic acid showed good interactions with Tamm–Horsfall protein having the least glide score of −2.35. The glide score, which distinguishes molecules based on their interacting ability, was calculated for the ligand. The least glide score or energy was more effective. The active compound showed good interaction with Tamm–Horsfall protein having the glide score of −5.465.

Our results coincides with Dinnimath and Jalalpure (2015) who observed the in silico antiurolithiatic screening of *Aerva lanata* L. isolated constituent. It showed glide scores of −4.37 and −4.11. The glide score, which distinguishes molecules based on their interacting ability, was calculated for the ligand. The least glide score or energy was more effective. The active compound 14-hydroxy-12-abietene-7-one showed good interactions with Tamm–Horsfall protein having the least glide score of −5.202.

The effect of Tamm–Horsfall protein isolated from urine of healthy subjects on calcium oxalate precipitation was studied in model systems of precipitation by Benkovic et al. (1995). Tamm–Horsfall protein was found to inhibit the growth of calcium oxalate in vitro monohydrate crystals and stimulate their aggregation. Both effects were enhanced by increase in the concentrations of Tamm–Horsfall protein and were most pronounced at the concentration of Tamm–Horsfall protein of 10 mg/l. Hydrogen bonding plays a major role in determining the specificity of intermolecular interactions. The active compound with least glide score clearly showed the ability to make maximum number of hydrogen bonds with the active site residues (Pandey and Ramos 2003).

Carvalho et al. (2002) screened the role of Tamm–Horsfall protein and uromodulin in calcium oxalate crystallization. Results indicate a different effect of Tamm–Horsfall protein may act on nucleation and inhibit crystal aggregation, while uromodulin may promote aggregation of calcium oxalate crystals. Comparison of melting point IR, ^1^HNMR, ^13^C NMR and mass spectral data obtained with the previously reported data for the diterpenoid abietane led to the conclusion that the compound isolated from aqueous bark extract of *Crateva magna* as 14-hydroxy-12-abietene-7-one. The glide score, which distinguishes molecules based on their interacting ability, was calculated for the ligand. The least glide score or energy was more effective. The active compound 14-hydroxy-12-abietene-7-one showed good interactions with Tamm–Horsfall protein having the least glide score of −5.202. The results exhibited the antiurolithiatic potential of bark extract of *Crateva magna* (Radha 2015).

Hamsa et al. (2013) has documented the molecular docking of natural compounds against NFkB p50/p65. The study revealed that ginkgetin, bilobetin and mesuaxanthone B exhibited the best binding reactions. Sahul et al. (2012) has also documented the utility of molecular docking in identifying the suitable plant compound stigmasterol from *Avicennia marina* plant against the VP28 envelope protein of WSSV. According to Piccagli et al. (2008) docking performance is predictive of a biochemical activity. They demonstrated it by the molecular docking simulation of a promising lead compound for the inhibition of NF-kappa b-p50 biological activity and modulation of the expression of the NF-kB regulated IL8 gene.

The availability of whole genome sequences has led to a new era in drug development, with bioinformatics often having the primary step in drug discovery programme today. Tamm–Horsfall protein (THP) is one of the main components of urinary protein origination from the kidney. The action of Tamm–Horsfall protein as an inhibitor or a promoter of calcium oxalate crystal growth and aggregation appears to depend on the degree of its aggregation, ionic strength of the medium and the glycoprotein concentration. At low ionic strength, urine Tamm–Horsfall protein acts as an inhibitor of calcium oxalate crystallization (Lopez et al. 1986). The inhibitory action is also pronounced at very low concentrations of Tamm–Horsfall protein (Scur and Robertson 1986). Moreover, THP becomes a promoter of CaOx aggregation due to a tendency to self-aggregation, which removes it from effective interaction with CaOx monohydrate crystals (Lopez et al. 1986).

## Conclusions

The treatment of urolithiasis is mainly considered with the dissolution of existing stones and preventing recurrence of stones. In the present study a triterpenoid compound 3-*O*-acetyl-11-keto-β-boswellic acid was obtained from aqueous root extract of *R. aquatica*. It was characterized by different techniques like IR, ^1^H NMR, ^13^C NMR and mass spectral data. Comparison of the obtained melting point of the isloated compound with the previously reported data for the triterpenoid boswellic acid led to the conclusion that the compound isolated from aqueous root extract of *R. aquatica*. Further by docking analysis the compound 3-*O*-acetyl-11-keto-β-boswellic acid interacted with Tamm–Horsfall protein. The interaction results exxhibits the ability of the compound towrds inhibition of calcium oxalate crystallization. The study encourages the utility of the compound for future drug discovery through advanced techniques.This can help the patients with urolithiatic difficulties without undergoing complex chemical based treatments. The herbal extracted compound can provide a healthy approach without any side effects as it is obtained from the nature and also reduced the burden on exploitation of herbs for treatments.
